# Distinct Cell Types With the Bitter Receptor Tas2r126 in Different Compartments of the Stomach

**DOI:** 10.3389/fphys.2020.00032

**Published:** 2020-02-07

**Authors:** Patricia Widmayer, Vanessa Partsch, Jonas Pospiech, Soumya Kusumakshi, Ulrich Boehm, Heinz Breer

**Affiliations:** ^1^Institute of Physiology, University of Hohenheim, Stuttgart, Germany; ^2^Experimental Pharmacology, Center for Molecular Signaling, School of Medicine, Saarland University, Homburg, Germany

**Keywords:** T2R, Tas2r, bitter sensing, brush cell, gastric groove, glandular units, enteroendocrine cells

## Abstract

Cells expressing bitter taste receptors (T2Rs or Tas2rs) in extraoral tissues are considered to be chemosensory cells mediating protective responses to potentially harmful or even antiinflammatory or antimicrobial compounds. In a previous study the activity of the *Tas2R143/Tas2R135/Tas2r126* cluster promoter in the stomach was monitored using a Cre-reporter mouse line. Reporter gene expression and *Tas2r126* mRNA were found in brush cells located at the distal wall of the gastric groove. In this study, we explored whether brush cells and epithelial cells of the stomach in fact contain the Tas2r126 receptor protein. Using immunohistochemistry, we demonstrate the presence of Tas2r126 immunoreactivity in different cell populations in the glandular stomach, in a subset of brush cells at the gastric groove and in unique glandular units as well as in certain enteroendocrine cells. In brush cells at the gastric groove, a strong immunofluorescence signal for the Tas2r126 receptor was observed at the most apical region of the cells, i.e., the microvillar tuft. In addition, we found a high density of Tas2r126-positive brush cells in the unique glandular units. These invaginations are located distally to the groove, open directly into the furrow and are enwrapped by smoothelin-immunoreactive muscles. In the corpus, Tas2r126 immunoreactivity was found in histamine-producing ECL cells and in ghrelin-producing X/A-like cells, the main enteroendcrine cells of this compartment. In the antrum, Tas2r126 labeling was observed in serotonin-storing EC cells and ghrelin cells, both representing only minor populations of enteroendocrine cells in this compartment. In conclusion, our data provide evidence for the presence of the Tas2r126 receptor protein in distinct cell types in the epithelium lining the mouse stomach which render the stomach responsive to agonists for bitter receptors.

## Introduction

Bitter compounds, such as secondary plant substances, strongly influence food intake (Fraenkel, 1959) as well as metabolic processes (Dotson et al., 2008; Vegezzi et al., 2014; Avau et al., 2015). In addition, several studies have shown their antiinflammatory or antimicrobial effects (Kaji et al., 2009; Prandi et al., 2013, 2018; Hariri et al., 2017) as well as their effect on hormone release in the gastrointestinal (GI) tract (Chen et al., 2006; Rozengurt et al., 2006; Jeon et al., 2008, 2011; Janssen et al., 2011; Kim et al., 2014). As a prerequisite for the induction of these characteristic effects, it is assumed that cells exist, which can sense such compounds and are positioned in distinct compartments of the GI tract.

Recognition of bitter compounds is based on a large family of G protein coupled receptors, the Tas2r receptors (Adler et al., 2000; Chandrashekar et al., 2000; Matsunami et al., 2000). The mouse genome comprises 35 genes encoding distinct Tas2R receptor types (Shi et al., 2003; Hayakawa et al., 2014; Li and Zhang, 2014). Meanwhile, there are several reports indicating that genes encoding receptors for bitter compounds are not only expressed in taste cells, but also in solitary cells interspersed in the epithelium lining the GI tract (see for review: Rozengurt, 2006; Sternini et al., 2008; Behrens and Meyerhof, 2011; Xie et al., 2018). However, very little is known about the expression of bitter receptors in the stomach. The current information is mainly based on PCR analyses (Wu et al., 2002, 2005; Vegezzi et al., 2014; Prandi et al., 2018); only recently immunohistochemical evidence was presented for the presence of TAS2R10 receptor protein in parietal and chief cells of human stomach (Liszt et al., 2017).

In a previous study, the activity of a promoter that controls the gene cluster *Tas2R143/Tas2R135/Tas2r126* was visualized by fluorescent marker protein (EGFP) expression in a transgenic reporter mouse line and revealed EGFP-labeled cells in the epithelium of the stomach (Liu et al., 2017). These were mostly located at the boundary between fundus and corpus and found to express DCLK1, a marker for brush cells, which are also called tuft, caveolated, multivesicular, or fibrillovesicular cells (Luciano and Reale, 1992). In fact, ∼15% of all DCLK1-positive brush cells were EGFP-positive; furthermore, in isolated brush cells only mRNA for the receptor gene *Tas2r126* was found (Liu et al., 2017). However, it remained elusive whether these cells indeed comprise the Tas2r126 receptor protein. Based on previous studies, brush cells at the gastric groove, a tissue fold at the boundary between fundus and corpus, are considered to be putative taste-like chemosensory cells (Höfer et al., 1996; Eberle et al., 2013). These cells are arranged in a palisade-like manner and express elements of the canonical taste signaling cascade, including gustducin (Höfer et al., 1996; Hass et al., 2007), PLCβ2 (Eberle et al., 2013) and TRPM5 (Kaske et al., 2007) as well as receptors for nutrients (Hass et al., 2010; Janssen et al., 2012; Eberle et al., 2014; Widmayer et al., 2015, 2019). Situated at a strategic position between the storage compartment fundus and the digestive compartment corpus, brush cells supposedly act as sensor cells monitoring the constituents of the luminal content and consequently influencing the regulation of gastric processes, such gastric motility, secretion and hormone release. Based on the results of RNA-seq analysis by Liu et al. (2017), we set out to analyze whether the Tas2r126 receptor protein is in fact present in gastric cells, to determine their localization in the stomach epithelium and to characterize the cell types which express the receptor Tas2r126.

## Materials and Methods

### Mice

Studies were performed with adult C57/BL6J and TRPM5-IRES-Cre/eR26-τGFP (Kusumakshi et al., 2015) mice. Animals were fed *ad libitum* with standard laboratory chow and had free access to water. For tissue preparations, animals were killed with carbon dioxide.

### Tissue Preparation

For immunohistochemistry and Western blotting, tongues and stomachs were removed and washed in ice-cold PBS (0.85% NaCl, 1.4 mM KH_2_PO_4_, 8 mM Na_2_HPO_4_, pH 7.4). For immersion fixation of the stomach, the fundus was cut off, the stomach opened along the greater curvature and washed with ice-cold PBS. For plane sectioning, the opened stomach was mounted on a piece of rubber and fixed with needles (Eberle et al., 2013). For longitudinal sectioning, a transverse strip of the entire stomach wall including the fundus, cardia, corpus and antrum was excised.

All tissues were fixed using 4% paraformaldehyde (in 150 mM phosphate buffer, pH 7.4) for 2 h. After fixation, tissues were cryoprotected by incubation in 25% sucrose overnight at 4°C. Then, tissues were embedded in Tissue Freezing Medium (Leica Microsystems, Bensheim, Germany) and quickly frozen on liquid nitrogen-cooled metal covers.

### Western Blotting

Total protein was extracted from pooled posterior papillae. 30 μg of the homogenate were loaded onto a 12.5% SDS-PAGE gel and transferred to a nitrocellulose membrane. The immunoblot was blocked with 6% milk powder in PBS buffer containing 0.1% Tween 20 (TBST) for 1 h and then probed with a rabbit anti-Tas2r126 antiserum (SAB signaling antibody, #44656, Baltimore, United States; rabbits were immunized with a synthesized peptide derived from human TAS2R41, the ortholog of mouse Tas2r126, predicting a species reactivity for human, mouse and rat) diluted 1:200 in 5% BSA in TBST overnight at 4°C. After washing in TBST, the blot was incubated with a goat anti-rabbit horseradish peroxidase (HRP)-conjugated IgG antiserum (Sigma-Aldrich), diluted 1:10000 in blocking solution and developed using the Amersham ECL Select kit (Thermo Fisher Scientific). The membrane was scanned using a C-DiGit blot scanner (LI-COR Biotechnology, Bad Homburg, Germany).

### Immunohistochemistry

Each tissue was serially cut into 6–8 μm thick sections. Cryosections were mounted onto Superfrost Plus microscope slides (Menzel-Gläser, Braunschweig, Germany) using a CM3050S cryostat (Leica Microsystems), air-dried for 1 h and stored at −20°C until use. For immunohistochemistry, cryosections underwent citrate-antigen-retrieval. Frozen sections were incubated in a sodium citrate buffer (10 mM sodium citrate, 0.05% Tween 20, pH 6.0) for 45 min at 4°C. Afterward, sections were immersed in the same sodium citrate buffer for 1 min at 100°C. Then slides were rinsed in PBS for 10 min and blocked in 0.3% Triton X-100 in PBS containing 10% normal donkey serum (NDS; Dianova, Hamburg, Germany) for 1 h at room temperature. After three changes of PBS, slides were incubated with the primary antisera overnight at 4°C. Antibodies were diluted in 0.3% Triton X-100 in PBS containing 10% NDS and used in the following dilutions: chicken anti-GFP [1:400; ab13970, Abcam, Cambridge, United Kingdom; van der Heijden et al. (2016)], rabbit anti-TRPM5 serum73 [1:600; purified antibody (AB-321); described in Kaske et al. (2007)], goat anti-COX-2 [1:200; sc-1747, Santa Cruz, Dallas, TX, United States; Widmayer et al. (2019)], rabbit anti-smoothelin [1:200, sc-28562, Santa Cruz; Frick et al. (2017)], goat anti-chromogranin A [1:200; sc-1488, Santa Cruz; Gerbe et al. (2011)], goat anti-HDC [1:20, sc-34458, Santa Cruz; Frick et al. (2017)], goat anti-ghrelin [1:800, sc-10368, Santa Cruz; Caminos et al. (2003)], goat anti-5-HT [1:3000; ab66047, Abcam, Cambridge, United Kingdom; Lund et al. (2018)], and goat anti-somatostatin [1:800, sc-7819, Santa Cruz; Frick et al. (2017)]. After washing in PBS, primary antisera were visualized using donkey anti-goat IgG (H + L) Alexa Fluor 488, Invitrogen (1:500; Fisher Scientific, Goteborg, Sweden), donkey anti-rabbit IgG (H + L) Alexa Fluor 568, Invitrogen (1:500; Fisher Scientific), and donkey anti-goat AMCA (1:200; Jackson, ImmunoResearch), diluted in PBS with 0.3% Triton X-100 containing 10% NDS for 2 h at room temperature. After a further three washes with PBS, tissue sections were counterstained with DAPI (1.0 μg/mL, Sigma-Aldrich) 1:1000. After incubation for 3 min at room temperature, sections were rinsed with double-distilled water and mounted in MOWIOL (10% polyvinyl alcohol 4-88 (Sigma-Aldrich), 20% glycerol in 1x PBS). No immunoreactivity was observed when the primary antisera were omitted.

### Cell Counting

Digital microscopic images of longitudinal sections were acquired at 40× magnification. For cell counts in the corpus and antrum, a continuous epithelial area of 2000 μm × 250 μm was defined starting at the edge of the proximal stomach and transitional junction from corpus to antrum, respectively. Counts were then expressed as cells per 0.5 mm^2^. To determine brush cell numbers in large invaginations, images of three consecutive longitudinal sections through the proximal stomach were acquired. Then, the average cell counts of immunopositive cells were determined. Cells were quantified as percentage of DAPI-labeled nuclei of immunoreactive cells. To assess the proportion of Tas2r126-expressing cells among enteroendocrine cells, the number of immunoreactive cells on 3–5 consecutive sections within an area of 355 μm × 265 μm was counted. The percentage and number of positive cells were expressed as a mean ± SD.

## Results

### Visualization and Characterization of Tas2r126-Immunoreactive Cells

**FIGURE 1:**
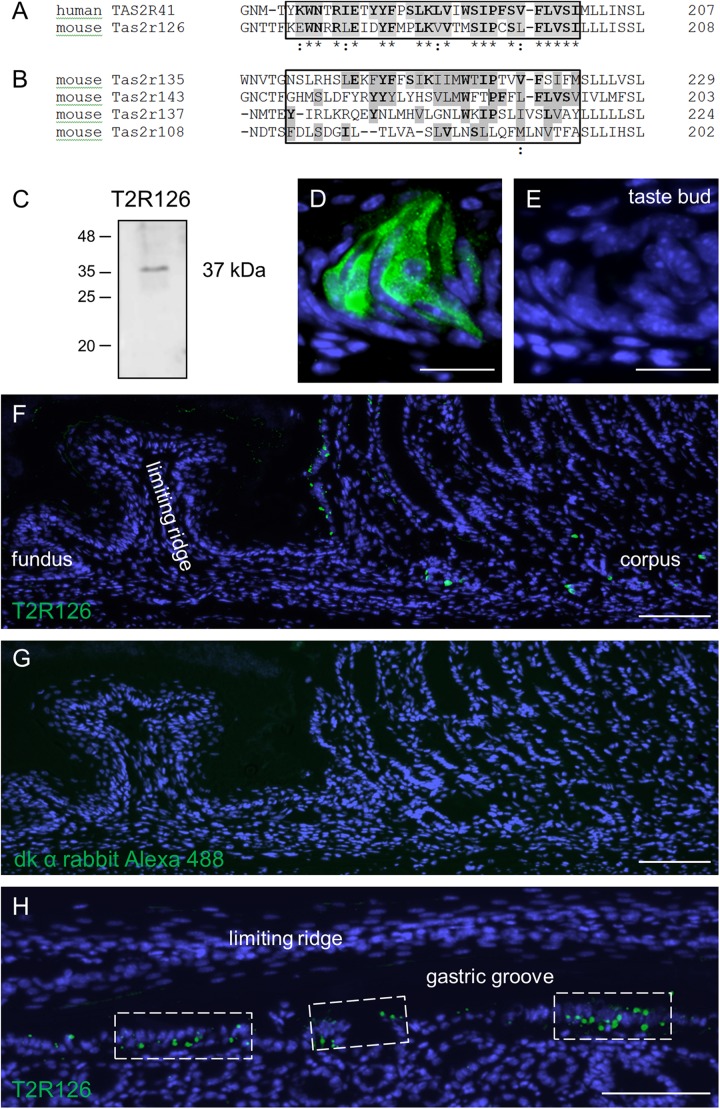
Sequence alignments and expression of the bitter receptor Tas2r126 assessed by Western Blot analyses and immunohistochemistry in mouse tongue and stomach. **(A)** Comparison of the sequence region for the one-to-one orthologs, human TAS2R41 and mouse Tas2r126, showing the sequence region used for the antiserum generation. Identical amino acids are highlighted in gray with bold letters, conservative replacements shaded in gray and divergent residues in white. The epitope alignment is marked in the box and indicates a considerable degree of sequence identity and similarity between TAS2R41 and Tas2r126. **(B)** Comparison of the corresponding sequence region of Tas2r subtypes which originate from the same phylogenetic clade and which are expressed in the stomach. There is a high degree of sequence diversity within the epitope region (boxed area) compared to TAS2R41, thus cross-reactivity with Tas2r subtypes other than Tas2r126 seems unlikely. Alignments were performed with the program Clustal Omega, EMBL-EBI. **(C)** Western blot analysis of whole cell lysates from posterior taste papillae revealed that the Tas2r126 antiserum recognized a band with an apparent molecular weight of ∼37 kDa corresponding with the expected size of 36.2 kDa. **(D)** In posterior taste buds the antiserum specifically labeled gustatory cells. **(E)** A consecutive section of the papilla probed with only the secondary antibody showed no labeling. **(F)** Immunohistochemistry on tissue sections through the whole stomach, longitudinally opened along the greater curvature, to analyze the aglandular and glandular gastric regions. In the fundus, no immunopositive cells were detected, whereas opposite the limiting ridge multiple Tas2r126-positive cells were labeled. In the corpus mucosa Tas2r126-immunoreactive cells were visible in the basal half of the invaginations. **(G)** A minus primary antiserum control of a consecutive section did not show any staining. **(H)** Analysis of a perpendicular section through the gastric ridge region showing multiple Tas2r126-positive cells in clusters. Scale bars **(D**,**E)**, 20 μm, **(F**–**H)**, 100 μm.

Previous experimental evidence for *Tas2r126* expression in the stomach is based on EGFP-positive epithelial cells in a Cre-reporter mouse line and RNA-seq analysis of isolated EGFP-positive cells (Liu et al., 2017). In order to analyze whether the receptor protein is in fact present in epithelial cells lining the stomach, we used an antiserum against Tas2r126 in immunological approaches. For this, we employed a commercially available antiserum raised against human TAS2R41 sharing an overall amino acid sequence identity of 69% to the mouse Tas2r126. The TAS2R41 epitope is localized in the region of the extracellular loop 2 and transmembrane domain 5 that shares a high number of identical and conserved amino acid residues with the mouse one-to-one ortholog (Figure 1A). To evaluate a potential cross-reactivity with related stomach-specific bitter receptors (Prandi et al., 2018), the relevant sequence motifs were compared. The alignment shows a high degree of diversity (Figure 1B) indicating that cross-reactivity is unlikely. Since no blocking peptide was provided by the supplier to prove the epitope specificity of the antiserum, we experimentally validated its specificity by performing Western blot analyses with preparations of posterior taste papillae. The result is depicted in Figure 1C and demonstrates that the antiserum recognized a protein at the expected size. We then probed tissue sections through the posterior region of the murine tongue with the Tas2r126 antiserum. As shown in Figure 1D, distinct cells in taste buds were specifically labeled; in contrast, sections incubated only with the secondary antibody were devoid of any staining (Figure 1E). Subsequently, tissue sections from the mouse stomach were analyzed. As depicted in Figure 1F, the epithelium of the aglandular fundus was devoid of any labeled cells. Instead, Tas2r126-immunolabeled cells were restricted to the glandular compartment of the stomach. Omission of the primary antiserum on an adjacent section shows the absence of any staining (Figure 1G). Particularly striking was the high density of labeled cells opposite of the limiting ridge, which surrounds the entire fundus/corpus border of the stomach as well as the esophageal orifice (Wattel and Geuze, 1978; Luciano and Reale, 1992). Beneath the limiting ridge large clusters of Tas2r126-positive cells were visible (Figure 1H). This clustering of the antiserum-labeled cells at the so-called gastric groove is reminiscent of the pattern of EGFP-positive cells in the Cre-reporter mouse line (Liu et al., 2017). In addition, we also found Tas2r126-immunopositive cells in the mucosal glands of the corpus (Figure 1F), where the labeled cells were mainly located in the basal half of the epithelium.

**FIGURE 2:**
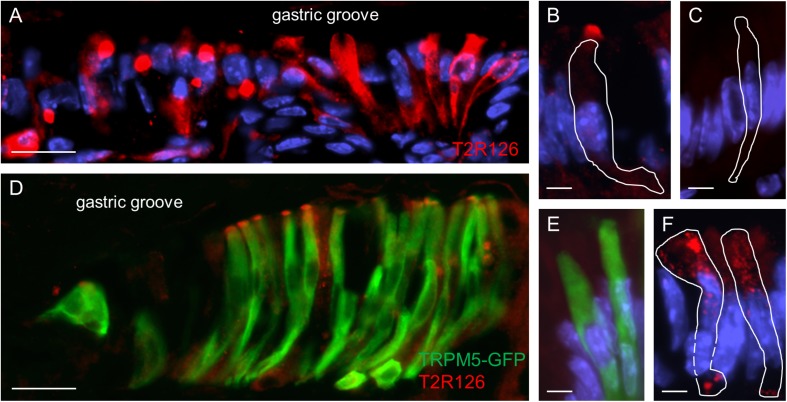
Expression of Tas2r126 in cells of the epithelial band at the gastric groove circumscribing the stomach. **(A)** Cells in the epithelial band displayed different staining characteristics. While in some cells the entire cell body was labeled, other cells showed a more punctate staining. **(B)** Several cells were characterized by a strong immunoreactivity at the most apical part; apparently the apical microvillar tuft of brush cells. **(C)** Omission of the primary antiserum served as negative control. **(D)** The identity as brush cells was confirmed analyzing TRPM5-IRES-Cre/eR26-τGFP mice; the τGFP immunostaining (green) is colocalized with Tas2r126 immunoreactivity (red) in a subset of brush cells. **(E)** Omission of the Tas2r126 antiserum resulted only in τGFP immunostaining (green). **(F)** Some columnar cells with a wide microvillar apex were also stained. Beneath the apical cell membrane, punctate staining was visible. Scale bars **(A**,**D)**, 20 μm, **(B**,**C**,**E**,**F)**, 5 μm.

Upon closer inspection of the labeled cells at the gastric groove a variety of staining patterns emerged. While in some cases the entire cell body was labeled, in other cases only a punctate staining was observed (Figure 2A). For some of the cells strong immunoreactivity was selectively visible at the apical region; the tuft-like labeling suggests that these cells are brush cells (Figure 2B). To test this hypothesis, we used TRPM5 as a brush cell marker and a transgenic mouse line allowing us to visualize TRPM5-expressing cells due to their endogenous GFP fluorescence (Kusumakshi et al., 2015). The results of double labeling experiments visualizing GFP and Tas2r126 immunoreactivity are depicted in Figure 2D. As can be seen, several of the TRPM5-positive cells showed a strong immunoreactivity for Tas2r126 at the apical region, probably at the microvilli. This result indicates that some of the Tas2r126-positive cells at the gastric groove are in fact brush cells. However, only a subset of the brush cells was Tas2r126-immunoreactive consistent with the observations by Liu et al. (2017). Additionally, some columnar cells also showed Tas2r126 immunoreactivity. These cells are characterized by a wide microvillar apex (Figure 2F). Controls omitting the Tas2r126 antiserum on sections from wildtype and transgenic mice resulted in negative staining (Figure 2C) and GFP-specific labeling (Figure 2E), respectively, thus indicating the specificity of the used antiserum.

### Tas2r126-Positive Cells in Large Invaginations at the Bottom of the Gastric Groove

**FIGURE 3:**
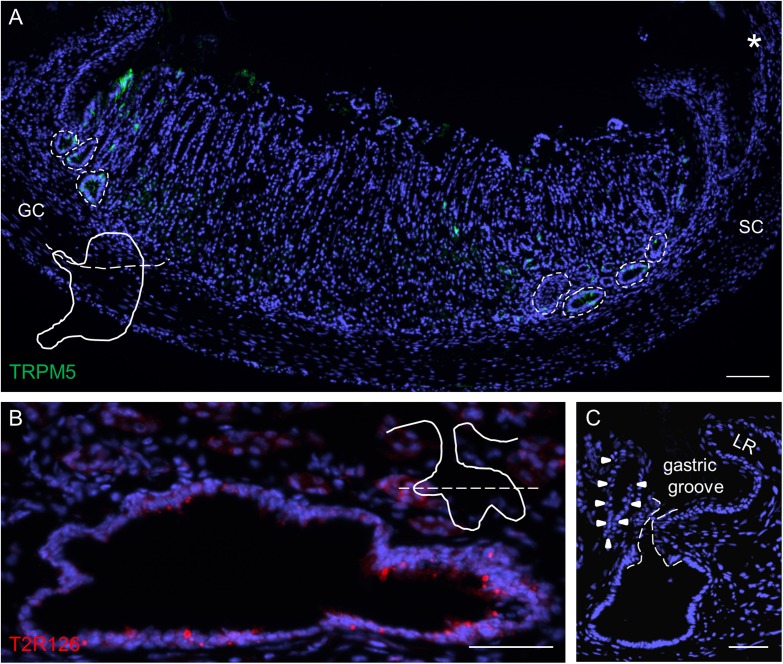
Analyses of Tas2r126-expressing cells in unique glandular units at the smaller and greater curvature adjacent to the gastric groove. **(A)** Distribution of large invaginations on stomach sections at the level of the esophagus orifice (asterisk). TRPM5 was used as marker for brush cells. In order to prepare the sections the stomach was cut along the dotted line as illustrated. **(B)** Large invaginations were often branched and fused into oval structures with a large lumen; the lining epithelium comprised numerous cells with Tas2r126 immunoreactivity (red). An example area at the smaller curvature is shown. The schematic drawing indicates the cutting level (dotted line). **(C)** Invaginations associated with the first gland (marked by arrow heads) which are located very close to the bottom of the gastric groove have an open connection to the gastric lumen. The connecting channel of the invagination is encircled by the dotted line. GC, greater curvature, SC, smaller curvature, LR, limiting ridge. Scale bars **(A)**, 100 μm, **(B**,**C)**, 50 μm.

Upon inspection of sections through the proximal edge of the corpus, we identified large invaginations that comprised a large number of brush cells (Figure 3A) and often also contained a high density of Tas2r126-labeled cells (Figure 3B). The invaginations are located in a narrow region, distally adjacent to the gastric groove at both the smaller and the greater curvature; this region corresponds to the so-called cardiac zone according to Wattel and Geuze (1978). This structure, called first gland, is considered to be a unique anatomical entity (O’Neil et al., 2017). The invaginations are often branched, almost ovoid or sometimes round in shape and have different sizes (small: 40 μm × 40 μm; large: 100 μm × 60 μm up to 240 μm × 160 μm). Those invaginations adjacent to the gastric groove open directly into this furrow (Figure 3C).

**FIGURE 4:**
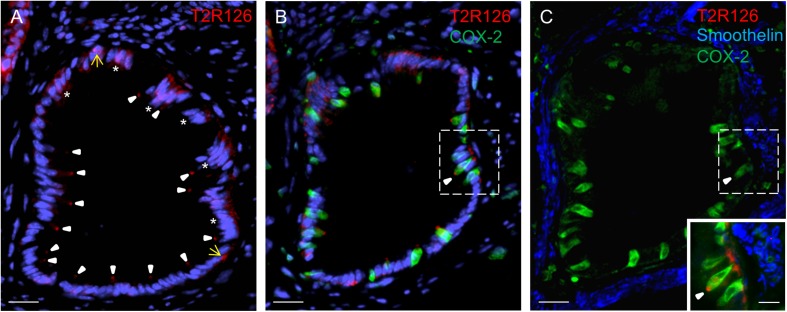
Cells with Tas2r126 immunoreactivity in glandular units at the bottom of the gastric groove enwrapped by smooth muscles **(A)** In these invaginations Tas2r126 labeling was found at the apex of brush cells (arrowhead) and basally located round cells (yellow arrow); furthermore groups of columnar cells (*) were labeled. **(B)** Brush cells with Tas2r126 immunoreactivity (red) contain the rate limiting enzyme for the synthesis of prostaglandin COX-2 (green). **(C)** A consecutive section was probed with a smoothelin antiserum (blue); the labeled structures surround the invagination so that smooth muscle cells are located next to the COX-2 positive brush cells (green). Inset: An overlay of the marked regions in **(B,C)** indicates that brush cells (green) with Tas2r126 immunoreactivity (red) are adjacent to smooth muscle cells (blue). Scale bars **(A**–**C)**, 20 μm, (inset), 10 μm.

Within these invaginations, we detected Tas2r126 labeling at the apex of brush cells, at the base of round cells and some columnar cells (Figure 4A). Quantification indicated that within the epithelium of these structures 57 ± 5.9% of all brush cells were also positive for Tas2r126 (*n* = 3 mice); whereas at the limiting ridge only ∼15% of the brush cells were found to express Tas2r126 (Liu et al., 2017). At the bottom of the gastric groove, the invaginations merged into the thick *lamina muscularis mucosae* that further extends deep into the limiting ridge (Luciano and Reale, 1992). To explore whether the Tas2r126-expressing brush cells might come in close contact with muscle fibers in this region, we performed immunolabeling for Tas2r126, COX-2 and smoothelin. Figure 4B shows the distribution of Tas2r126-expressing brush cells within an invagination, which is enwrapped by muscle fibers (Figure 4C). At higher magnification it becomes apparent that some Tas2r126-immunoreactive brush cells are indeed located in close vicinity to smooth muscle cells (Figure 4C, inset).

### Characterization of Tas2r126-Positive Cells in Mucosal Glands

**FIGURE 5:**
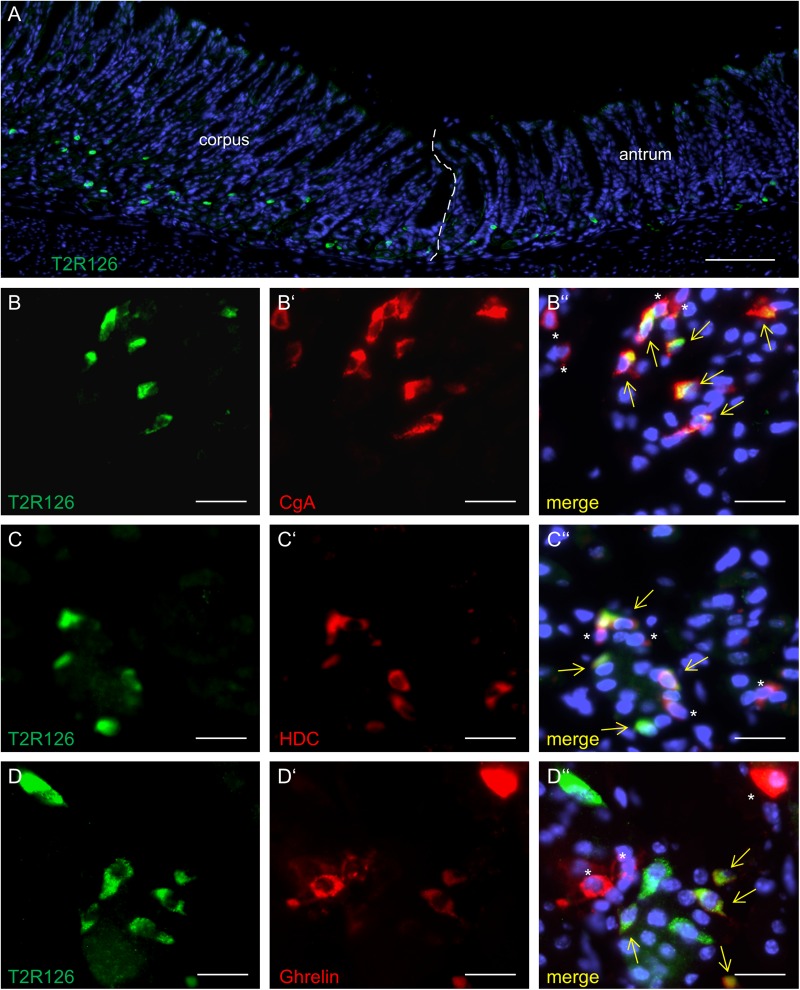
Molecular phenotypes of cells with Tas2r126 immunoreactivity in the glandular regions corpus and antrum. **(A)** On sections of the transition region between the corpus and antrum it was apparent that the antrum mucosa contained only very few Tas2r126-positive cells compared to the corpus mucosa. **(B**–**D)** Double immunohistochemical analyses of the corpus mucosa. **(B)** Labeling for Tas2r126 (green) and CgA (red) indicates that several CgA-positive cells were also positive for Tas2r126. **(C)** Visualization of histamine-producing cells (red) by an antiserum for histidine decarboxylase (HDC) revealed that several were also immunopositive for Tas2r126 (green). **(D)** A subset of X/A-like cells, identified by ghrelin (red), was also immunoreactive for Tas2r126 (green). Yellow arrows point to co-labeled cells (merge). Asterisks mark hormone-producing cells devoid of Tas2r126 staining. Scale bars **(A)**, 100 μm, **(B**–**D)**, 10 μm.

**FIGURE 6:**
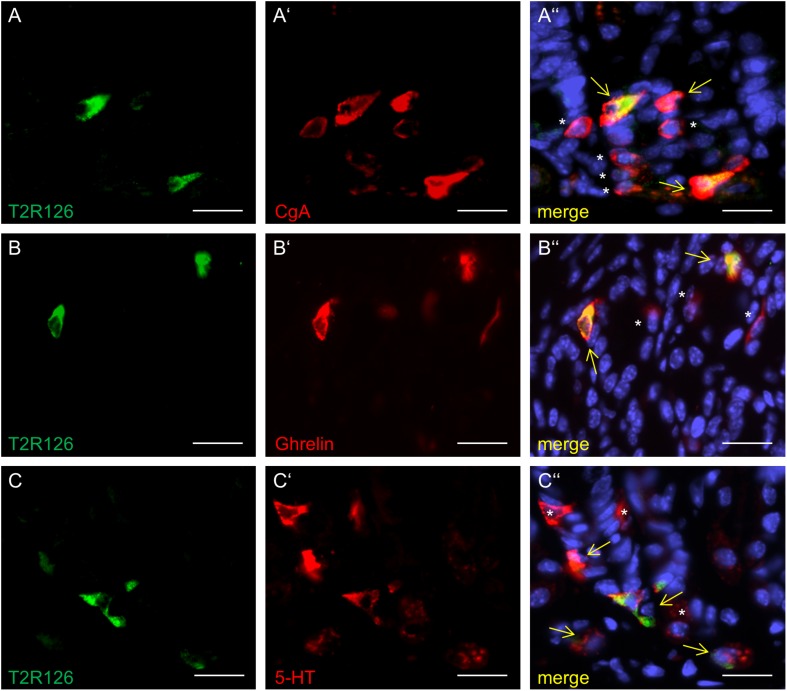
Distinct enteroendocrine cell types in the mouse antrum express Tas2r126. **(A)** Numerous CgA-positive EECs (red) were found to be immunoreactive for Tas2r126 (green). **(B)** Cells which were double labeled for Tas2r126 (green) and ghrelin (red) were also found in the antrum. **(C)** Dual immunostaining showed the presence of Tas2r126 immunoreactivity (green) in serotonin-producing EC cells, identified by 5-HT (red). Yellow arrows point to co-labeled cells (merge). Asterisks mark hormone-producing cells devoid of Tas2r126 staining. Scale bars **(A**–**C)**, 10 μm.

Analyzing corpus and antrum for Tas2r126-immunoreactive cells revealed that labeled cells were present in both compartments, albeit at quite different densities (Figure 5A). Whereas 60 ± 9.6 labeled cells per 0.5 mm^2^ were counted in the corpus (*n* = 3 mice), the antrum only contained 17.3 ± 3.5 cells per 0.5 mm^2^ (*n* = 3 mice). Based on the localization of the cells at the lower half of the invaginations the Tas2r126-immunoreactive cells in the corpus and antrum glands may be enteroendocrine cells (EECs). To test this hypothesis, double labeling experiments were performed using the antiserum against Tas2r126 in combination with an antiserum against CgA, a general marker for EECs. The results depicted in Figure 5B indicate that among the CgA-positive cells several were immunoreactive for Tas2r126; in fact, this applied to 52 ± 8.4% of the CgA cells (*n* = 3 mice). In order to further characterize the molecular phenotype of the Tas2r126-expressing EECs in the corpus, we used an antiserum against HDC, the key enzyme involved in synthesis of histamine, as well as antisera against ghrelin, serotonin, and somatostatin. In the corpus glands, subpopulations of Tas2r126-positive cells were labeled with antisera against HDC and ghrelin, respectively (Figures 5C,D). The analyses of numerous preparations revealed that the proportion of EEC cells, which were positive for Tas2r126, decreased from proximal to distal. In antral glands, only 21 ± 9.3% of CgA-positive cells were positive for Tas2r126 (*n* = 3 mice) (Figure 6A); subsets were found to be co-labeled for serotonin (5-HT) or for ghrelin (Figures 6B,C). In both compartments, Tas2r126-immunoreactive somatostatin-producing D cells were only rarely seen (data not shown).

## Discussion

In the present study, we have identified distinct cell types in the gastric epithelium expressing a bitter receptor protein; thus, confirming and extending the results of previous studies, which demonstrated the presence of mRNA for bitter receptors in epithelial cells of the stomach (Wu et al., 2002; Vegezzi et al., 2014; Liu et al., 2017; Prandi et al., 2018). Expression of the Tas2r126 receptor in distinct cell types enables the stomach to sense certain bitter compounds and elicit adequate responses. Based on the results of PCR experiments one may assume that additional types of bitter taste receptors are present in the stomach. In fact, a recent study identified a set of distinct Tas2r receptor types that was expressed in the stomach (Prandi et al., 2018). In view of possible implications it will be of great interest to further explore whether individual combinations of receptor types may be expressed in distinct cell types or in cells at certain positions. This would allow defined responses to various bitter compounds and may explain diverse effects of bitter compounds on stomach functions, such as gastric emptying (Glendinning et al., 2008), antral contractility, stimulation of ghrelin release (Janssen et al., 2011, 2012; Wang et al., 2019) and induction of gastric acid secretion (Liszt et al., 2017, 2018).

The presence of Tas2r126 protein in distinct epithelial cells of the stomach is consistent with a functional role of Tas2r126 agonists in regulating gastric processes. Deorphanization studies of mouse Tas2r by heterologous expression using a large variety of compounds revealed that the receptor type Tas2r126 has an intermediate molecular receptive range; compounds which activate Tas2r126 all share a phenyl ring and include arbutin and salicin (Lossow et al., 2016). Interestingly, arbutin is considered to have antimicrobial effects (Ma et al., 2019) and salicin to have antiinflammatory effects (Li et al., 2015). Thus, cells equipped with a functional Tas2r126 receptor might be involved in gastro-protective processes.

The presence of the bitter receptor Tas2r126 in a subset of brush cells, which are arranged in a continuous band beneath the limiting ridge as demonstrated in this study and by Liu et al. (2017), is particularly interesting in view of previous findings demonstrating that brush cells at the gastric ridge comprise receptor types for short chain fatty acids (Eberle et al., 2014) as well as for long chain fatty acids (Widmayer et al., 2015, 2019). An obvious question in this context is whether the bitter receptor-expressing brush cells also contain short and/or long chain fatty acid receptors or whether they constitute separate populations. Brush cells with a broad receptor expression profile may operate as multisensory cells capable to respond to a variety of chemical constituents in the luminal content, whereas distinct sets of bush cells with different chemosensory properties could point to region-specific functions. Moreover, it is unclear whether all brush cells at the gastric ridge have the enzymatic capacity to produce the candidate messenger compounds, such as nitric oxide, prostaglandins and acetylcholine (Kugler et al., 1994; Eberle et al., 2013; Widmayer et al., 2019) which could operate as paracrine mediators between brush cells and adjacent effector cells. The high proportion of brush cells with Tas2r126 immunoreactivity in the large invaginations at the proximal edge of the corpus is a striking observation. Although the functional implication is unknown, it is interesting to note that these invaginations constitute, together with the so-called first gland, a unique glandular unit enriched in brush and mucus cells, which are considered to be a source of the mucin MUC4 (O’Neil et al., 2017).

The arrangement of Tas2r126 cells at the border between the reservoir compartment fundus and the digestive compartment corpus with secretory epithelial cells might point to an involvement of brush cells in defense mechanisms by controlling the activity of secretory cells. Interestingly, this zone is separating the luminal content of the two compartments as well as in controlling the transfer from the aglandular fundus into the glandular part of the stomach. The ingested food stored in the fundus is probably separated by an adequate mucus barrier generated by the numerous mucus-containing glands (Lee et al., 1982) and columnar cells secreting the TFF1 (unpublished results); the activity of both may be controlled by the sensing of brush cells. In this context, it is conceivable that ingested secondary plant compounds, such as arbutin, which is present in various berries and activate the Tas2r126 receptor, might induce such effects.

Stimulation of Tas2r126 in brush cells thus might result in the activation of the canonical downstream components of the taste signal transduction (Wong et al., 1996; Rössler et al., 1998; Liu and Liman, 2003), since all functional elements of the canonical taste signaling cascade are expressed in the stomach, including gustducin, PLCβ2, TRPM5 (Höfer et al., 1996; Hass et al., 2007; Kaske et al., 2007; Eberle et al., 2013). According to the proposed mechanism, it is conceivable that an activation of bitter receptors in brush cells will lead to the dissociation of the βγ-dimer from the α-subunit gustducin and an activation of PLCβ2 that in turn generates IP_3_ that opens IP_3_R channels to release calcium. Elevated calcium levels then may activate TRPM5 channels leading to a calcium influx and consequently the release of signaling molecules. Such paracrine messengers may in turn induce the secretion of mucins, TFF1 or antimicrobial peptides from the surrounding epithelial cells. In fact, studies by Lee et al. (2014, 2017) demonstrated that in the airways bitter agonist-induced activation of brush cells leads to a secretion of β-defensin 1.

Some of the invaginations close to the gastric groove open into the furrow and are enwrapped by smooth muscles (Wattel and Geuze, 1978). The close vicinity of brush cells and smooth muscle cells may suggest some paracrine influence of brush cells on the contraction state of muscles. The interaction between brush cells and smooth muscles could for example be involved in tightly closing the stomach opening (Montedonico et al., 1999) and in guiding ingested food into the fundus. Furthermore, for brush cells in the small intestine it has recently been shown that they trigger IL-25-mediated type 2 immune responses upon a parasite and bacterial infection (Gerbe et al., 2016; Howitt et al., 2016; von Moltke et al., 2016; Nadjsombati et al., 2018). It is currently unknown whether brush cells in the stomach are also involved in the regulation of local immune circuits. Based on the afore mentioned examples, Tas2r126-expressing brush cells might be considered as sentinel cells of the stomach, however, the mechanisms associated with their proposed protective role still awaits elucidation.

In this report, we provide evidence that a subpopulation of ghrelin-producing X/A-like cells and histamine-storing ECL cells in the corpus contain the Tas2r126 receptor. This observation is in line with transcriptome analysis of sorted ghrelin cells demonstrating enrichment of *Tas2r126* RNA when compared to non-fluoresent cells (Engelstoft et al., 2013). Ghrelin cells are typically closed type cells which are assumed to be activated via the blood stream. However, it has recently been shown that salicin, a potent activator of the Tas2r126 receptor, affects the secretion of ghrelin as well as gastric emptying and food intake (Janssen et al., 2011). Conversely, the bitter compounds denatonium benzoate (DB) or phenylthiocarbamide (PTU) evoked opposite effects (Janssen et al., 2011). These differences support the notion that additional Tas2R receptor types are active in the stomach, possibly in different subpopulations of cells. In histamine- and ghrelin-producing cells the stimulation with Tas2r126 agonists may elicit an elevation of intracellular calcium and thus trigger the release of hormones. Indeed, recent studies showed that certain bitter agonists stimulate the secretion of ghrelin from human and mouse cells (Janssen et al., 2011; Wang et al., 2019). Activation of ghrelin cells via bitter receptors may involve gustatory α-subunits, since both gustducin and transducin were found to be expressed in subpopulations of mouse ghrelin cells and the lack of gustducin affected acyl ghrelin (Janssen et al., 2012).

In conclusion, distinct cell types with the receptor Tas2r126 are located at characteristic positions in the stomach and may operate as a specialized surveillance system monitoring the luminal content for defined constituents. Moreover, these cells might convey the sensed information via paracrine signaling onto gastric effector systems, adapting the motor and secretory activities according to the changing composition of the luminal content.

## Data Availability Statement

All data generated or analyzed during this study are included in this published article.

## Ethics Statement

The animal study was reviewed and approved by the institutional and national guidelines for the care and use of laboratory animals according to the Society of Laboratory Animals (GV-SOLAS) were followed. The work was approved by the Committee on the Ethics of Animal Experiments at the Regierungspräsidium Stuttgart (V318/14 Phy) and the University of Hohenheim Animal Welfare Officer (T125/14 Phy, T126/14 Phy). All experiments complied with the Principles of Animal Care, publication no. 85-23, revised 1985, of the National Institutes of Health and with the current laws of Germany.

## Author Contributions

PW conceived and planned the study, analyzed the data, and wrote the manuscript. VP, JP, and PW conducted the experiments. HB contributed to the conception of the study, interpreted the results, and wrote the manuscript. UB and SK reviewed the manuscript and provided critical feedback. All authors refined and approved the final version of the manuscript.

## Conflict of Interest

The authors declare that the research was conducted in the absence of any commercial or financial relationships that could be construed as a potential conflict of interest.

## Abbreviations

CgA: chromogranin A
COX-2: cyclooxygenase 2
DAPI: 4′, 6-diamidino-2-phenylindole
DCLK1: double cortin-like kinase
EC: enterochromaffin cell
ECL: enterochromaffin-like cell
HDC: histidine decarboxylase
IP_3_: inositol-1,4,5-trisphosphate
IP_3_R: inositol-1,4,5-trisphosphate receptor
PLC β 2: phospholipase C β 2
TFF1: trefoil factor 1
TRPM5: transient receptor potential cation channel, subfamily M, member 5
5-HT: 5-hydroxytryptamin
